# Lipopolysaccharides with Acylation Defects Potentiate TLR4 Signaling and Shape T Cell Responses

**DOI:** 10.1371/journal.pone.0055117

**Published:** 2013-02-04

**Authors:** Anna Martirosyan, Yoichiro Ohne, Clara Degos, Laurent Gorvel, Ignacio Moriyón, Sangkon Oh, Jean-Pierre Gorvel

**Affiliations:** 1 Aix-Marseille University UM 2, Centre d’Immunologie de Marseille-Luminy, Marseille, France; 2 INSERM U 1104, Marseille, France; 3 CNRS UMR 7280, Marseille, France; 4 Baylor Institute for Immunology Research, Dallas, Texas, United States of America; 5 Institute for Tropical Health and Departamento de Microbiología y Parasitología, Universidad de Navarra, Pamplona, Spain; National University, Costa Rica

## Abstract

Lipopolysaccharides or endotoxins are components of Gram-negative enterobacteria that cause septic shock in mammals. However, a LPS carrying hexa-acyl lipid A moieties is highly endotoxic compared to a tetra-acyl LPS and the latter has been considered as an antagonist of hexa-acyl LPS-mediated TLR4 signaling. We investigated the relationship between the structure and the function of bacterial LPS in the context of human and mouse dendritic cell activation. Strikingly, LPS with acylation defects were capable of triggering a strong and early TLR4-dependent DC activation, which in turn led to the activation of the proteasome machinery dampening the pro-inflammatory cytokine secretion. Upon activation with tetra-acyl LPS both mouse and human dendritic cells triggered CD4^+^ T and CD8^+^ T cell responses and, importantly, human myeloid dendritic cells favored the induction of regulatory T cells. Altogether, our data suggest that LPS acylation controlled by pathogenic bacteria might be an important strategy to subvert adaptive immunity.

## Introduction

Dendritic cells (DC) play a key role in initiating and controlling the magnitude and the quality of adaptive immune responses [1], [2]. Upon exposure to microbial stimuli, DC undergo a maturation process characterized by an increased formation of MHC–peptide complexes, the up-regulation of co-stimulatory molecules, chemokine receptors and cytokine production [1], [2], [3]. Cytokines produced by DC play a key role in determining the type of generated CD4^+^ helper T cell (T_H_) responses leading to T_H1_, T_H2_ or T_H17_ responses [1], [2]. Moreover, DC play a pivotal role in the control of central tolerance and the induction of immune tolerance in the periphery. The ability of DC to induce tolerance depends on several parameters such as their maturation stage, anti-inflammatory and immunosuppressive agents, the nature of microbial stimuli, and the tissue microenvironment. In addition to deleting T cells, tolerogenic DC induce the differentiation and proliferation of T cells with regulatory/suppressive functions known as regulatory T cells (T_reg_) [4].

Lipopolysaccharide (LPS) is an important virulence factor of Gram-negative bacteria responsible for septic shock in mammals. LPS is the major molecule of the bacterial outer membrane and can be massively released into the host during the course of infection [5], [6]. LPS consists of the O-polysaccharide chain, the oligosaccharide core region and the lipid A. Typical LPS such as those of *E. coli* and most enteric bacteria express a lipid A composed of a bisphosphorylated glucosamine disaccharide carrying two amide- and two ester-linked acyl and hydroxyacyl chains. Additional acyloxyacyl chains are commonly present, resulting in penta or hexa-acyl lipid A, the dominant molecular lipid A species in most wild type enterobacteria [7], [8]. It has been shown that variations of structural arrangements of lipid A such as a reduction in the number of charges or the number of acyl chains or a change in their distribution or saturation degree result in a dramatic reduction in endotoxicity. For instance, the synthetic precursor tetracyl lipid IVa has been described as a non-endotoxic molecule and proposed as an antagonist of hexa-acyl endotoxic LPS [9], [10]. Moreover, some pathogens like the yersiniae modulate the degree of acylation of the lipid A depending upon the environmental conditions. Most notably, growth at 37°C causes *Yersinia pestis* to synthesize tri- and dominant tetra-acyl lipid A, with no hexa-acyl and only small amounts of penta-acyl molecules. Since these bacteria move from 20–25°C to 37°C when transmitted from the flea to the mammal host, *Y. pestis* express tetra-acyl lipid A which displays low immunostimulatory properties in mammals. This change has been described as a mark of pathogen adaptation to the host environment [7].

In this study, we investigated the relationship between lipid A acylation and the immunostimulatory properties of LPS in the context of mouse and human DC activation. We show that LPS with acylation defects described as not endotoxic are capable of inducing a strong and early TLR4-dependent cell activation. This leads to the activation of the proteasome machinery and the degradation of newly synthetized pro-inflammatory cytokines. Mouse and human DC activated by tetra-acyl LPS trigger CD4^+^ and CD8^+^ T cell responses. Moreover, human DC activated by LPS with acylation defects display a semi-mature phenotype and induce high levels of regulatory T cells (T_reg_).

## Materials and Methods

### Ethics Statement

Animal experimentation was conducted in strict accordance with good animal practice as defined by the French animal welfare bodies (Law 87–848 dated 19 October 1987 modified by Decree 2001–464 and Decree 2001–131 relative to European Convention, EEC Directive 86/609). All animal work was approved by the Direction Départmentale des Services Vétérinaires des Bouches du Rhône (authorization number 13.118). INSERM guidelines have been followed regarding animal experimentation (authorization No. 02875 for mouse experimentation).

Blood from healthy adult donors were collected at the Baylor Hospital Liver Transplant Clinic (Dallas, TX) after obtaining written informed consent. This study, including the consent form, was approved by the Institutional Review Board (IRB) of the Baylor Research Institute (BRI) (Dallas, TX). Any medical issue during blood collection from healthy donors was written and reported to the IRB at BRI.

### Lipopolysaccharides

The methods used in the extraction, purification and characterization of the LPS used in this study have been described previously (Lapaque et al, 2006). Briefly, *Y. pestis* KIM6, *E. coli* MLK3 and its lipid A mutants MLK53 *htrB* ^−^ (lauroyl-transferase), MLK 1067 *msbB* ^−^ (miristoyl-transferase) and MLK 986 *htrB* ^−/^ *msbB* ^−^ were grown at the appropriate temperature, crude LPS obtained by the phenol-water method and then purified to remove traces of contaminant lipids and lipoproteins. The degree of lipid A acylation was determined by nano-electrospray ionization time-of-flight mass spectrometry (ESI-TOF-MS) (Lapaque et al, 2006). For all experiments, LPS variants have been used at the concentration of 100 ng/ml. Lipid Iva was purchased from PeptaNova.

### Antibodies and Reagents

The primary antibodies used for immunofluorecence microscopy were: mouse FK2 antibody (anti-mono- and polyubiquitinylated conjugates) (Enzo Life Science), affinity purified rabbit “Rivoli” antibody against murine I-A, NF-kB subunit p65/ReiA (Santa Cruz), CD11c (Bolegend). Pam2CSK4 was purchased from InvivoGen to activate DC. Antibodies used for flow cytometry included APC-CD11c (1 in 100), FITC-CD40 (1 in 50), FITC-CD80 (1 in 50), PE-CD86 (1 in 400), PE-IA-IE (MHC class II) (Pharmingen) (1 in 800), as well as PB-CD8 (1 in 200), A700-CD45.2 (1 in 300), APC-CD44 (1 in 400), PE-Cy7-CD25 (1 in 1500), APC-CD62L (1 in 400) (BD Biosciences and eBiosciences). For intracellular labeling of cytokines, IL-12 (p40/p70)-PE and TNF-α PE monoclonal antibodies (1 in 100)(Pharmingen) were used. The Aqua Dead Cell Stain (Invitrogen) was used to eliminate dead cells. Ovalbumine (OVA) was purchased from EndoGrade with purity>98% and endotoxin concentration <1EU/mg. SIINFEKL peptide was purchased from Schafer-N. Human mDC were sorted from PBMC of blood from healthy donors using lineage cocktail-FITC (BD Biosciences), CD123-PE (BD Biosciences), CD11c-APC (Biolegend), HLA-DR-Quantum Red (Sigma). Human mDC were stained with CD86-PE, CD83-FITC, CD40-APC and HLA-DR-PB (eBiosciences or Biolegends). 7-AAD was used to exclude dead cells. For intracellular labelling IL13-APC, INF-γ-PE-Cy7, IL-17-PE and Granzyme B-APC antibodies were used. Isotype matched controls were used appropriately. Alexa Fluor 647 conjugated phospho-specific antibodies were used for Phospho flow experiments on human IL-4 DC and were all from BD Biosciences. Akt(S478), Btk(Y557)/Itk(Y511), CREB(S133)/ATF1(S63), ERK1/2(T202/Y204), IRF-7(S477/S479), Lck(Y505), NF-κB p65(S529), PLC-γ1 (Y783), PLC-γ2 (Y759), p38 MAPK(T180/Y182), b-Catenin (S45), SHP-2(Y542), Src(Y418), SLP-76(Y128), S6(S235/S236), STAT1(Y701), STAT1(S727), STAT3(Y705), STAT3(S727), STAT4(S693), STAT5(S694), STAT6(Y641), 4EBP1(T36/T45), Zap70(Y319)/Syk(Y352), JNK(T183/Y185).

### Mice and Cells

C57Bl/6 mice from Jackson Laboratory and OT-I, OT II TCR transgenic mice on C57Bl/6 background were used. C57BL/6, *Tlr4* ^−/−^ and *Tlr2* ^−/−^ mice were maintained at the CIML animal house, France. Mouse bone marrow-derived DC (BMDC) and macrophages (BMDM) were prepared from 7–8 week-old female C57BL/6 mice as previously described (Lapaque et al, 2006).

### Human DC

Human IL-4 monocyte-derived DC were generated from Ficoll-separated PBMC from healthy volunteers. Monocytes were enriched from the leukopheresis according to cellular density and size by elutriation as per manufacturer’s recommendations. For DC generation, monocytes were resuspended in serum-free Cellgro DC culture supplemented with GM-CSF and IL-4. Blood myeloid DC (HLA-DR^+^CD11c^+^CD123^−^Lin^−^) were sorted from fresh PBMC using FACSAria (BD Biosciences). Naïve CD4^+^ and CD8^+^ T cells (CD45RA^+^CD45RO^−^) (purity>99.2%) were purified by FACS-sorting.

### Immunofluorescence Microscopy

For immunofluorescence microscopy, 2×10^5^ stimulated BMDCs on coverslips were fixed in 3% paraformaldehyde at RT for 15 min, washed twice in PBS 1X and processed for immunofluorescence labelling. To stain NF-κB, mouse BMDCs and BMDMs were permeabilized with PBS 1X 1% saponin (for 10 min at RT) and then saturated with PBS 1X 2% BSA (for 1 h at RT). CD11c (1 in 100), NF-kB subunit p65/ReiA (1 in 250) and MHC II (1 in 300) were used as primary antibodies. After staining, samples were examined on a Zeiss LSM 510 laser scanning confocal microscope for image acquisition. Images were then assembled using Adobe Photoshop 7.0. Quantifications were done by counting at least 300 cells in 3 independent experiments.

### Flow Cytometry

To analyse mouse BMDC maturation, 2×10^5^ cells were stimulated and stained with antibodies for classical activation markers. Appropriate isotype antibodies were used as controls. After staining, cells were washed with PBS 2% FCS, then PBS 1X and fixed in 1.5% paraformaldehyde before analysis on a FACScalibur cytometer (Becton Dickinson). Cells were always gated on CD11c for analysis and 100,000 CD11c+ events were collected from each sample. For the intracellular staining of IL-12 and TNF-α in mouse BMDCs, BD Cytofix/Cytoperm and BD Perm/Wash buffers were used. At least 100.000 events were collected on FACSCanto II (BDBiosciences). For mouse CD4 and CD8 T cell assays, viable cells were analyzed for the decrease of CFSE (proliferation) and the expression of CD25, CD44 and CD62L (diluted in PBS 1X EDTA 2 mM). Human mDC or IL4-DC activation was analyzed by checking the surface expression of maturation markers CD40, CD83, CD86, HLA-DR after 16 h or 72 h of cell treatment with LPS variants, respectively. Flow cytometry analysis was performed using the FlowJo software. Histograms were drawn from and median fluorescence intensity values were determined on gated populations. At least 100,000 events were collected on FACSCanto II (BDBiosciences) or FACSAria (BDBiosciences).

### Cytokine Measurement

Murine IL-12 and TNF-α were quantified in culture supernatants of stimulated DC by sandwich enzyme-linked immunosorbent assays (ELISA) according to the manufacturer’s protocol (Abcys). Human cytokine (IL-6, TNF-α, and IL-12p40) were determined using the BeadLyte cytokine assay kit (Upstate, MA).

### Immunoblotting

30 µg of cell lysates were subjected to SDS-PAGE PAGE and, after transfer to nitrocellulose, the membrane was probed with a polyclonal antibody against phospho-S6 or S6 (Cell Signaling Technology) or an anti-actin antibody. Blots were subjected to enhanced chemiluminescence detection (ECL, PIERCE).

### Quantitative RT-PCR

Total RNA was isolated with Trisol reagent, was reverse transcribed and analyzed by real-time quantitative PCR using the Power SYBR Green PCR Master Mix (Applied Biosystems). All reactions were performed in triplets. Data were acquired on a 7500 Fast Real-Time PCR system (Applied Biosystems) and were normalized to the expression of *actin* mRNA transcripts in individual samples. For a given real-time qRT-PCR sample, the RNA expression level was calculated from cycle threshold (Ct). In our analysis, given gene expression is shown as mean normalized expression (MNE) relative to the expression of β-actin. The following primers were used for qPCR amplification:RT β-actin (sense): GACGGCCAGGTCATCACTATTG, RT β-actin (anti-sense): CAAGAAGGAAGGCTGGAAAAGA, p35 sense : 5′ctcctgtgggagaagcagac3′, p35 anti-sense: 5′acagggtgatgggctatctga3′, p40 sense:5′CACACTGGACCAAAGGGACT3′p40 anti-sensereverse: 5′ATTATTCTGCTGCCGTGCT3′, TNF-α sense: 5′CATCTTCTCAAAATTCGAGTGACAA3′TNF-α, anti-sense : 5′TGGGAGTAGACAAGGTACAACCC3′. 3 independent experiments were done and one representative is shown.

### *In vitro* Antigen Presentation Assays

BMDC (3000 cells) were incubated overnight in 96-well culture plates either with media or OVA. T cells obtained from the lymph nodes and the spleen of OT-I and OT-II Rag-2^−/−^ mice were purified with the T cell enrichment kit from Dynal following manufacturer’s instructions. For CD4 and CD8 T cell proliferation assays, purified T cells were labeled with 10 µM carboxyfluorescein diacetate succinimidyl ester (CFSE from Invitrogen) for 10 min at 37°C. OT-II and OT-I cells (20000 cells) were added to BMDC that had been stimulated for 8 h with different LPS and then washed. The proliferation of OT-I and OT-II T cells was assessed after 3 days of co-culture by flow cytometry. The cells were washed and stained with anti-CD4 and anti-CD8 antibodies for identification. For CD4 and CD8 T cell activation assays, purified T cells were co-cultured with BMDC previously stimulated for 8 h with different LPS. After 3 days, the expression of surface markers such as CD25, CD44 and CD62L was analyzed by flow cytometry to study the cellular activation level.

### Co-culture of OT-II T cells with BMDC

CD4^+^ T cells were isolated from the spleen of OT-II *Rag-2* ^−/−^ mice using a CD4^+^ T cell isolation kit (Dynal; Invitrogen). Purity was determined by staining with CD4, CD5, and TCR Vα2. A total of 3 × 10^3^ BMDC stimulated for 8 h with different LPS were co-cultured with 2 × 10^4^ OT-II *Rag-2* ^−/−^ T cells in the presence of ovalbumin, ovalbumin (257–264) peptide (0.06 µg/mL) and of TGF-β (1 ng/mL) as indicated. After 5 days of culture, the expression of Foxp3 and CD25 was evaluated.

### Human CD4+ and CD8+ T cell Responses

5×10^3^ blood mDC were co-cultured with CFSE-labeled allogeneic naïve CD4+ T and CD8+ T cells (1–2×10^5^). The DC/T ratio was 1∶1000 and 1∶20, respectively. Cell proliferation was tested by measuring CFSE-dilution on day 6. On day 7, the production of intracellular cytokines (INF-γ, IL-17, IL-13) and Granzyme B were analyzed after 6 h of T cell stimulation by PMA and Ionomycine, in the presence of Brefeldin A. Cells were stained for analysis by flow cytometry using different fluorochrome-conjugated antibodies.

### Phospho-flow Analysis with Fluorescent Cell Barcoding (FCB)

Monocyte-derived IL4 DC were generated as previously described. Briefly, human monocyte were enriched with human monocyte enrichment kit without CD16 depletion (Stemcell Technologies, Canada) and suspended in CellGro DC medium (CellGenix, Germany) with GM-CSF and IL-4. On day 6, cells were washed and resuspended at 1 million/mL in RPMI supplemented with 2 mM L-Glutamine, 1 mM Sodium pyruvate, 1X non essential amino acid, 50 µM b-ME, and 10 mM HEPES +10% FBS, and then cultured for 2 h in a CO2 incubator. Cells were stimulated with different LPS (100 ng/ml) for 2, 5, 10, 30, 60, and 180 min. Equal amount of medium was used for stimulation control. All samples were immediately fixed by adding PFA (final 1.6%) for 10 min at RT. Fixed cells were centrifuged and washed once with PBS, and then permeabilized with ice-cold Methanol (500 µl/1 million cells) for 10 min at 4°C. Two dimension FCB was performed according to the previous report [11]. Pacific Blue-NHS and Alexa Fluor 488-NHS (Invitrogen, Carlsbad, CA) were added to each condition of cells at 0.02, 0.08, 0.32, 1.0, 3.0 µg/ml or 0.05, 0.2, 0.8, 3.0 µg/ml, respectively. Each sample has a unique combination of dyes with different concentrations. After 30 min on ice, barcoded cells were washed three times with PBS+0.5% BSA and combined into one tube. Combined barcorded cells were stained with Alexa Fluor 647 conjugated phospho-specific antibodies for 30 min at RT. Cells were washed two times with PBS+0.5% BSA. For purified anti-phospho-JNK antibody, cells were stained with secondary anti-rabbit DyLight 649 (Jackson Immunoresearch, West Grove, PA) for 30 min at RT and washed two times. Samples were immediately analyzed with FACS CantoII (BD Biosciences, San Jose, CA). Fold changes of phosphorylation were visualized as a Heatmap. The MFI of LPS-stimulated samples were normalized with medium-stimulated samples.

### Statistical Analysis

All experiments were carried out at least 3 independent times and all the results correspond to the means ± standard errors. Statistical analysis was done using two-tailed unpaired Student's *t* test. Significance was defined when *P* values were <0.05.

## Results

### Structural Modifications of LPS Affect Cytokine Secretion by DC

**Figure 1:**
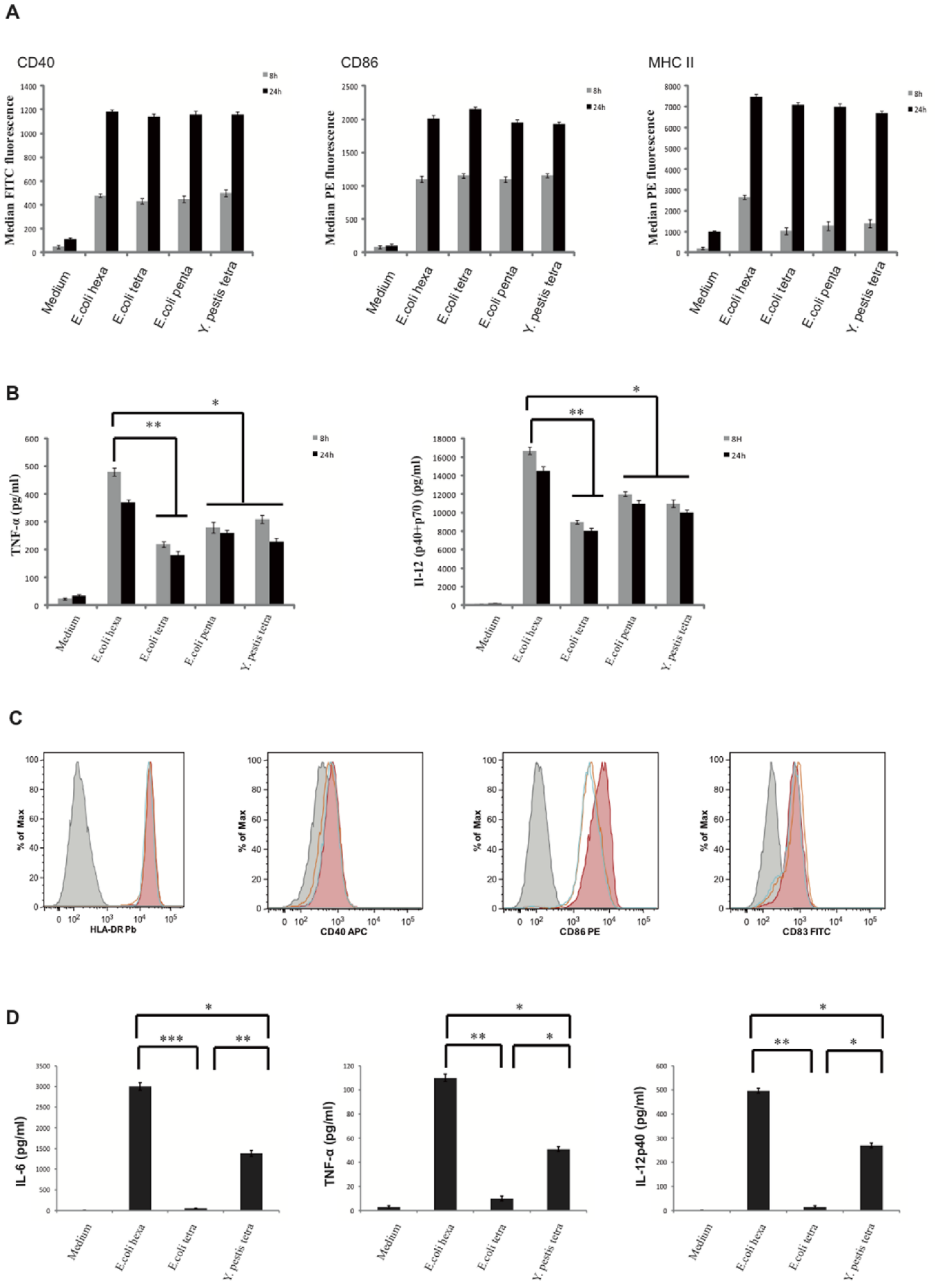
LPS with acylation defects induce semi-mature mouse and human dendritic cells. Mouse BMDC were stimulated for 8 h (in grey) and 24 h (in black) with medium, *E. coli* LPS (either hexa-acyl, penta-acyl or tetra-acyl) and *Y. pestis* tetra-acyl LPS. All LPS were used at the concentration of 100 ng/ml. MHC II and co-stimulatory molecules up-regulation on the cell surface was measured by flow cytometry (A) and cytokine secretion was determined by ELISA (B). Data represent means ± standard errors of at least 5 independent experiments, **p<0.01, *p = 0.01 to 0.05. Human blood mDC were stimulated overnight with medium (in grey), hexa-acyl *E. coli* LPS (in red), tetra-acyl *E. coli* LPS (in blue) and *Y. pestis* tetra-acyl LPS (in orange). Surface expression of HLA-DR, CD83, CD40 and CD86 was analyzed by flow cytometry (C) and cytokine levels in the culture supernatants were measured by Luminex (D). Experiments were performed on 4 different donors. The data for one representative are shown. ***p<0.001, **p<0.01, *p = 0.01 to 0.05.

We used an array of LPS (Table 1) differing in lipid A acylation to study their activation properties in mouse bone marrow-derived dendritic cells (BMDC) and bone marrow-derived macrophages (BMDM). In addition to the classical wild type hexa-acyl LPS purified from *E. coli* MLK strain, we used LPS from *E. coli* MLK mutants (*msbB*-, *htrB*- and *msbB*−/*htrB*- double mutant) that produce mostly penta-acyl and tetra-acyl lipid A (Table 1) or LPS purified from *Y. pestis* KIM grown at 37°C (mainly composed of tri- and tetra-acyl lipid A with small amounts of penta-acyl and hexa-acyl molecules, Table 1). All LPS variants induced a BMDC maturation characterized by an up-regulation of the surface expression of major histocompatibility complex MHC-II and co-stimulatory molecules (CD40, CD86) (Figure 1A). However, significant lower levels of secreted TNF-α and IL-12 were detected in DC stimulated by tetra-acyl LPS purified from *E. coli* MLK (*msbB*−/*htrB*-) double mutants or LPS purified from *Y. pestis* compared to DC stimulated with wild type *E. coli* hexa-acyl LPS (Figure 1B). Moreover, the LPS variants did not induce any IFN-α secretion (not shown). While comparing the activities of LPS variants, we have also performed a dose-response study (not shown). Cell treatment by 1 ng/ml of LPS triggered DC activation, which reached a plateau at the highest concentration (100 ng/ml). The same differences in terms of cytokine secretion were observed when cells were treated both with 100 ng/ml and 10 ng/ml of different LPS (not shown). Similarly, in BMDM activated by tetra-acyl LPS, TNF-α secretion was strongly decreased compared to BMDM incubated with hexa-acyl LPS (Figure S1) as previously observed in macrophage cell lines [8], [9], [10].

**Table 1:** Characteristics of LPS.

Bacterial strain (relevant genetic features) a	Proportions of lipid A species (molecular mass)
*E.coli* MLK3	>90% hexaacyl (1823.3 Da); traces of penta and tetraacyl.
*E.coli* MLK53 (*htrB-*)	rough-LPS; pentaacyl lipid A deficient in C12 oxyacyl of 3-OH-C14 acyl at GlcN C2′ (1615.1 Da)
*E.coli* MLK 1067 (*msbB-*)	rough-LPS; >90% pentaacyl (1587.0 Da); tetraacyl traces
*E.coli* MLK986 (*msbB*-, *htrB*-)	rough-LPS; 29% pentaacyl (1643.0 Da); 54% tetraacyl (1404.8 Da); and 17% triacyl (1178.6 Da)
*Y. pestis* KIM	rough-LPS, 9% hexaacyl (1797.2 Da); 10% pentaacyl; 40% tetraacyl (1404.8 Da); 7% arabinosamine- tetraacyl (1535.9 Da); 30% triacyl (1178.6 Da)

a All are rough-type LPSs.

We then tested the ability of tetra-acyl LPS (referred as purified either from *E. coli* MLK *msbB*−/*htrB*- double mutant or *Y. pestis* grown at 37°C) to induce human blood myeloid DC (mDC) activation (Figure 1C and D). Hexa-acyl and tetra-acyl LPS induced a similar up-regulation of classical cell surface activation markers (HLA-DR, CD40, CD86, and CD83) (Figure 1C). However, mDC treated with tetra-acyl LPS secreted lower levels of IL-12, IL-6 and TNF-α than those stimulated by hexa-acyl LPS (Figure 1D). Tetra-acyl LPS from *Y. pestis,* which contains small amounts of hexa-acyl LPS had a stronger capacity to trigger IL-12, IL-6 and TNF-α secretion (p<0.01) than LPS purified from *E. coli* (*msbB*-, *htrB*-) double mutant (devoid of hexa-acyl LPS) (Figure 1D, Table 1). Together, our data show that structural modifications of LPS induce an intermediate phenotype of maturation in mouse and human DC characterized by high levels of MHC-II and co-stimulatory molecule expression, but low levels of pro-inflammatory cytokine secretion.

### Tetra-acyl LPS Induce a TLR4-dependent DC Activation

LPS recognition by host cells is mediated through the Toll-like receptor 4 (TLR4/MD2/CD14) receptor complex [12]. To determine the contribution of TLR4 in the cell activation induced by LPS with acylation defects, BMDC derived from *Tlr4* ^−/−^, *Tlr2* ^−/−^ and wild type mice were treated with the LPS variants. No activation was observed in *Tlr4* ^−/−^ mice-derived BMDC stimulated either by hexa-acyl or tetra-acyl LPS (p<0.001), as measured by the secretion of TNF-α (Figure S2A). In addition, TLR2 was not implicated in DC activation induced by the different LPS (Figure S2B), showing that LPS preparations were not contaminated by lipoproteins.

**Figure 2:**
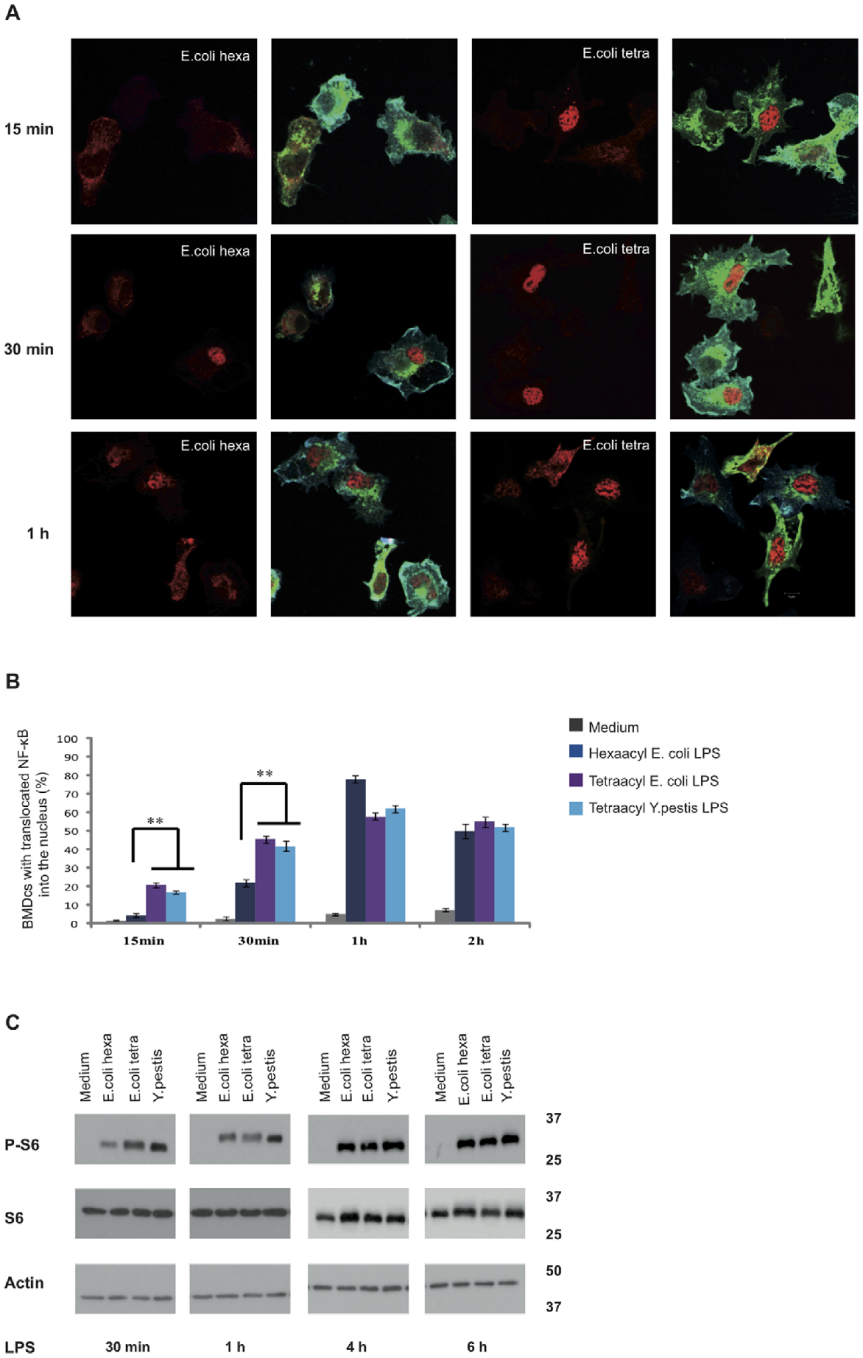
Tetra-acyl LPS induce the activation of TLR4-dependent molecular pathways involved in mouse DC maturation. BMDC were activated with medium (grey), *E. coli* hexa-acyl LPS (dark blue), *E. coli* tetra-acyl LPS (purple) or *Y. pestis* tetra-acyl LPS (light blue) for 15 min, 30 min, 1 h and 2 h. NF-κB translocation was analyzed by confocal microscopy(A). Cells were fixed and stained for CD11c (in blue), MHC-II (in green) and NF-κB subunit p65/RelA (in red). The percentage of BMDC with translocated NF-κB into the nucleus was quantified (B). BMDC were stimulated for 30 min, 1 h, 4 h and 6 h with medium or different LPS. Cell lysates were subjected to SDS-PAGE and, after transfer to nitrocellulose, the membrane was probed with the antibodies against phospho-S6 (Ser235/236), S6 and an anti-actin antibody (C). Data represent means ± standard errors of at least 4 independent experiments, **p<0.01.

The measurement of DC viability following treatment with different LPS showed that both hexa-acyl and tetra-acyl LPS induce a very low percentage of dead cells (0.93%) (not shown). We next tried to understand if the decrease of pro-inflammatory cytokine secretion in BMDC activated by tetra-acyl LPS was related to a defect in signal transduction. It has been shown that NF-κB translocation is a key event in LPS-induced TLR4 signalling [13]. Under unstimulated conditions, NF-κB is kept in the cytosol as an inactive form. Under hexa-acyl LPS stimulation NF-κB is translocated into the nucleus where it can bind to several gene promoters [13], [14]. After 15 and 30 min of cell stimulation, tetra-acyl LPS induced a significant (p<0.01) stronger NF-κB translocation than hexa-acyl LPS (Figure 2A and B). Similar results were observed in macrophages (Figure S3A and B).

Since the activation of the mammalian target of rapamycin (mTOR) pathway has been implicated in DC maturation [16], we then analyzed the phosphorylation of the ribosomal protein S6, one of downstream elements of the TLR4 pathway. Compared to hexa-acyl LPS, tetra-acyl LPS induced a stronger S6 phosphorylation at 30 min post-cell activation (Figure 2C). No difference for S6 phosphorylation was observed at later time points either by hexa-acyl or tetra-acyl LPS (Figure 2C). These data show for the first time that LPS with acylation defects induce an early and strong activation of the TLR4-dependent signalling pathway in mouse DC and macrophages.

**Figure 3:**
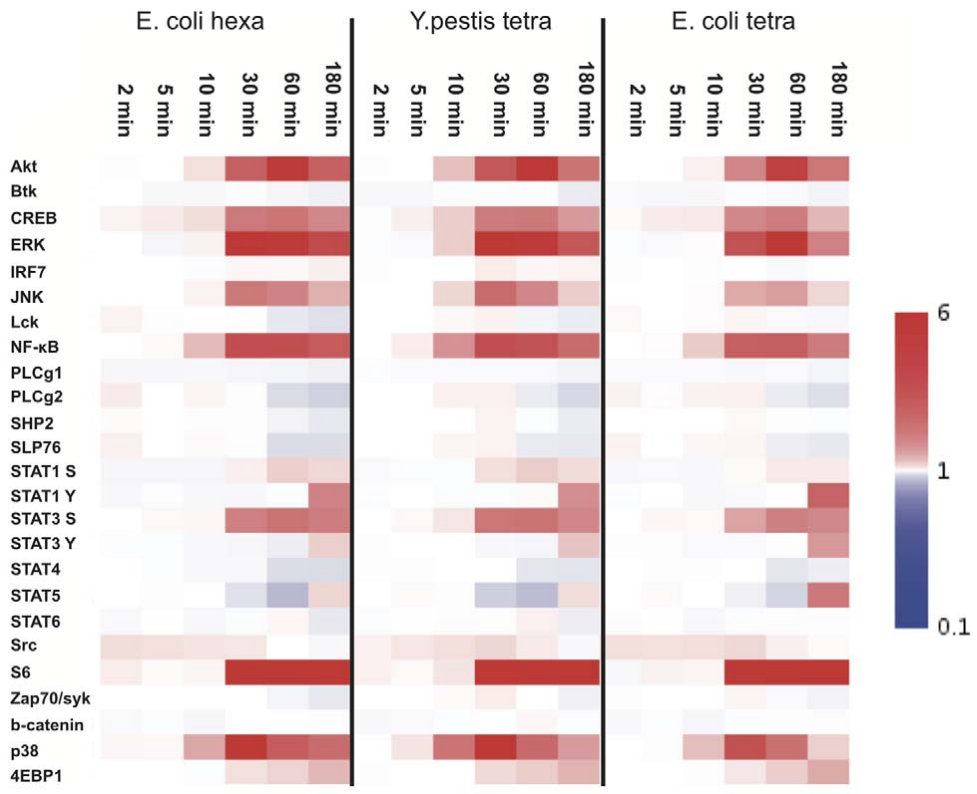
Phospho-flow analysis of human IL-4 DC stimulated by LPS. Human IL-4 DC were activated by different LPS for 2 min, 5 min, 10 min, 30 min, 60 min and 180 min. A phospho-flow analysis using fluorescent cell barcoading was performed in order to assess the phosphorylation levels of molecules involved in TLR4 signaling. The heatmap visualization of phosphorylation changes is shown. The median fluorescent intensity (MFI) of stimulated cells is normalized by MFI of medium stimulated cells. Colored bar on the right shows the levels of fold changes. Experiments were performed on 4 different donors. The data for one representative are shown.

We extended this study to human monocyte-derived IL-4 DC (Figure 3) by using the phospho-flow technology. Fluorescent cell barcoding (FCB) was applied to analyze many conditions simultaneously, using a collection of several anti-phosphorylated proteins [11]. All LPS variants LPS were equally able to increase the phosphorylation levels of several signaling molecules including MAPKs (ERK, p38, JNK), Akt-mTOR pathway molecules (Akt, 4EBP1, S6), and some transcription factors (CREB, NFkB p65) (Figure 3). Interestingly, although the patterns of phosphorylated molecules were same between LPS variants, the kinetics and strength of the phosphorylation changes were slightly different with several molecules (Figure 3). *Y. pestis* LPS could induce phosphorylation more rapidly, while LPS mutant caused phosphorylation more slowly and weakly than *E. coli* LPS in some molecules, especially in Akt, p38 and NFkB (Figure 3). These results suggest that as *E. coli* hexaacyl LPS, *Y. pestis* LPS and *E. coli* LPS mutant could act as an agonist to TLR4 pathway. However, structural differences in lipid A region may modify the LPS binding capacity to the receptor, leading to changes in activation potential. It should be also noted, *E. coli* LPS mutant enhanced tyrosine phosphorylation in STAT1, 3, 5 at later time point more potently than others (Figure 3). Taken together, LPS variants seem to activate the same signaling pathway with different activation potential that may affect the output and quality of immune responses induced by DC.

Thus, LPS purified from *E. coli* MLK (*msbB*-, *htrB*-) double mutant and *Y. pestis* were able to trigger TLR4-dependent signalling in human DC, in agreement with data obtained on mouse BMDC (Figure 2).

Altogether these data show that LPS with acylation defects act as agonists to the TLR4 pathway and efficiently induce signal transduction in mouse and human DC.

### Tetra-acyl LPS Induce an Early Synthesis of Pro-inflammatory Cytokines followed by their Proteasome-dependent Degradation

**Figure 4:**
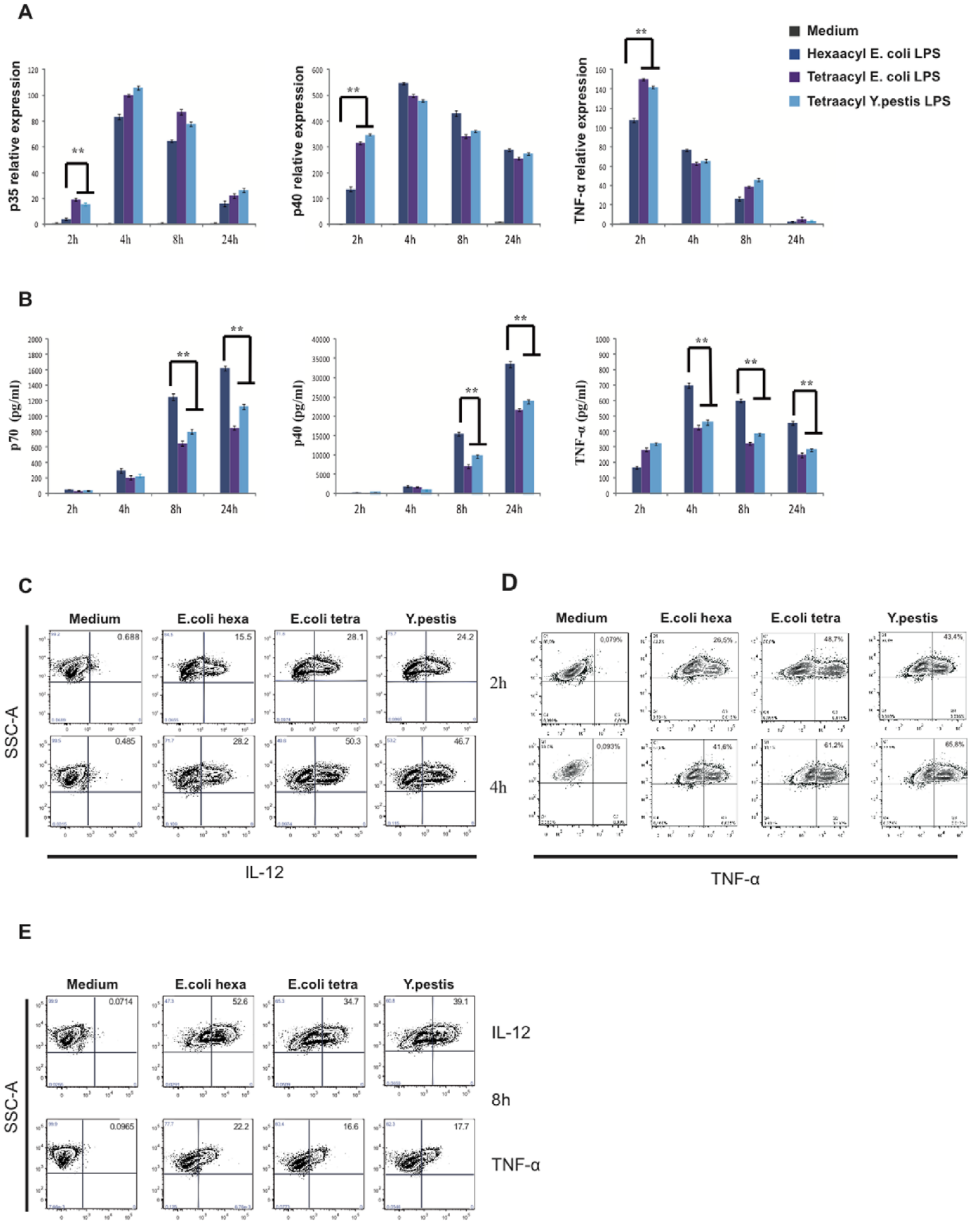
Kinetics of synthesis of pro-inflammatory cytokines. (A) BMDC were stimulated for 2 h, 4 h, 8 h or 24 h with medium (grey), *E. coli* hexa-acyl LPS (dark blue), *E. coli* tetra-acyl LPS (purple) or *Y. pestis* tetra-acyl LPS (light blue). Total RNA was purified from cell lysates, reverse transcribed and the amount determined by real-time quantitative PCR. Primers were used for qPCR amplification of *actin* (control), *p35, p40* and *TNF-α* genes. 3 independent experiments were done and one representative is shown, **p<0.01. (B) The secretion levels of IL-12p70, IL-12p40 and TNF-α were determined by ELISA. Data represent means ± standard errors of at least 4 independent experiments, **p<0.01. (C, D) BMDC were treated for 2 h and 4 h with medium, *E. coli* LPS (either hexa-acyl or tetra-acyl LPS) and *Y. pestis* tetra-acyl LPS. The intracellular synthesis of IL-12 (p40+p70) in (C) and TNF-α in (D) was analysed by flow cytometry. (E) The intracellular IL-12 and TNF-α production was studied in BMDC activated for 8 h with LPS variants. At least 3 independent experiments were performed and one representative is shown.

We then investigated whether the decrease of pro-inflammatory cytokine secretion in BMDC activated by tetra-acyl LPS was due to a defect in cytokine synthesis (transcription/translation). BMDC were activated with different LPS and quantitative RT-PCR used to analyse gene expression. In BMDC treated by tetra-acyl LPS an earlier and stronger transcription of *tnf-α*, *p35* and *p40* genes was observed (Figure 4A) compared to BMDC treated by hexa-acyl LPS. Therefore, the decrease of pro-inflammatory cytokine secretion observed in Figure 4B cannot be attributed to transcriptional defects.

**Figure 5:**
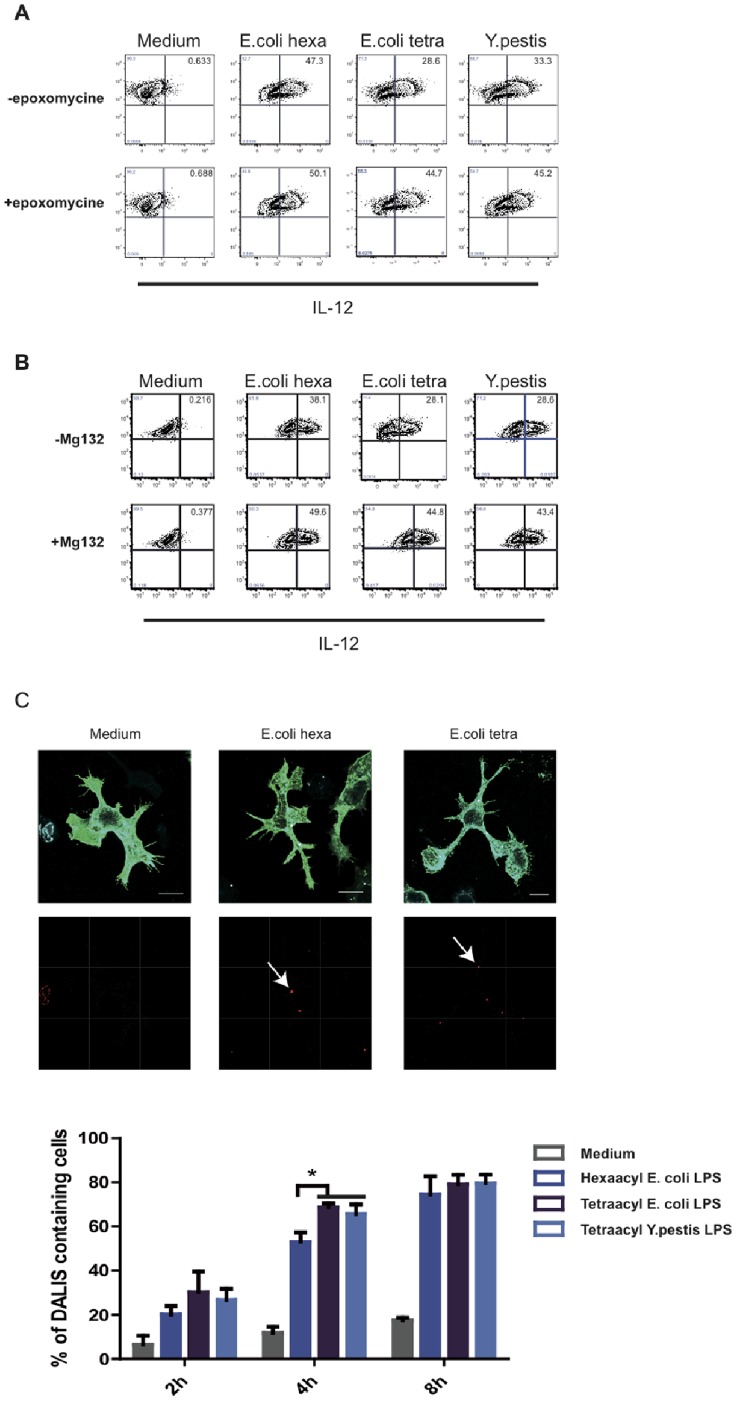
Tetra-acyl LPS induce a degradation of IL-12 by the proteasome machinery in DC. BMDC were activated for 8 h with LPS variants in the presence or the absence of proteasome inhibitors such as epoxomycine (A) and Mg132 (B). The intracellular IL-12 (p40+p70) synthesis was then analysed. At least 3 independent experiments were performed and one representative is shown. (C) BMDC were activated for 2 h, 4 h, 8 h and 24 h with LPS variants and labelled with anti-MHC II(green), anti-CD11c (blue) and FK2 (red) antibodies to detect DALIS (white arrows). Quantification of the percentage of DC with DALIS at 2 h, 4 h and 8 h post-incubation with medium or post-stimulation with the different LPS. Quantifications were done by counting at least 300 cells in 3 independent experiments. Data represent means ± standard errors of at least 3 independent experiments, *p = 0.01 to 0.05.

We next investigated whether the defect in cytokine secretion by DC stimulated with tetra-acyl LPS was due to a change in protein translation (Figure 4C and D). BMDC were incubated with the different LPS in the presence of brefeldin A to block the secretion of newly synthesized cytokines. Intracellular levels of IL-12 and TNF-α were analysed by flow cytometry. LPS with acylation defects induced significant higher TNF-α and IL-12 synthesis at 2 h and 4 h post-stimulation compared to hexa-acyl LPS (Figure 4C and D). However, at 8 h post-stimulation, the level of intracellular cytokines was lower in DC treated with tetra-acyl LPS than in DC treated by hexa-acyl LPS (Figure 4E). It has been shown that glucose or energy deprivation, calcium homeostasis perturbation or elevated synthesis of secretory proteins induce an alteration of the Endoplasmic Reticulum (ER) homeostasis [15], [16]. This leads to the disruption of protein folding, the accumulation of unfolded proteins and ER stress response or unfolded protein response (UPR) to restore ER normal function. One of the major components of UPR is the degradation of misfolded proteins by the proteasome (ER associated degradation, ERAD) [15], [16]. We therefore determined if the decrease of cytokine secretion observed in DC activated by tetra-acyl LPS could be due to a proteasome-mediated degradation of newly synthesized cytokines (Figure 5). Epoxomycine (Figure 5A) or Mg132 (Figure 5B) proteasome inhibitors were used in BMDC treated by the different LPS for 8 h and intracellular the IL-12 expression was analysed. As expected, in the absence of proteasome inhibitors the level of intracellular IL-12 expression was lower in tetra-acyl LPS-treated DC than in hexa-acyl LPS-treated DC. However, in the presence of proteasome inhibitors DC treated with tetra-acyl LPS levels of intracellular IL-12 were similar to those expressed by DC treated with hexa-acyl LPS (Figure 5A and B). We then studied the ubiquitinylation of proteins following DC activation by different LPS. It has been shown that upon inflammatory stimulation, DC accumulate newly synthesized ubiquitinylated proteins in large cytosolic structures. These DC aggresome-like induced structures (DALIS) are transient and require continuous protein synthesis [16]. Mouse DC treated with LPS variants underwent maturation and displayed MHC II surface localization as well as DALIS formation (Figure 5C). However, after 4 h of tetra-acyl LPS treatment, the percentage of DALIS-containing cells was significantly higher as compared to cell stimulated by hexa-acyl LPS (Figure 5C). At 24 h, the number of DALIS decreased, consistent with the transient DALIS expression previously demonstrated in the process of DC maturation (not shown) [16]. These data strongly suggest that tetra-acyl LPS induce a degradation of IL-12 by the proteasome machinery in DC. It is therefore tempting to hypothesize that LPS with acylation defects could induce an ER stress in DC activating the proteasome machinery. This will lead to the down-regulation of cytokine intracellular levels and consequently to a decrease of their secretion.

### LPS with Acylation Defects Induce Antigen-specific CD8^+^ and CD4^+^ T cell Responses

**Figure 6:**
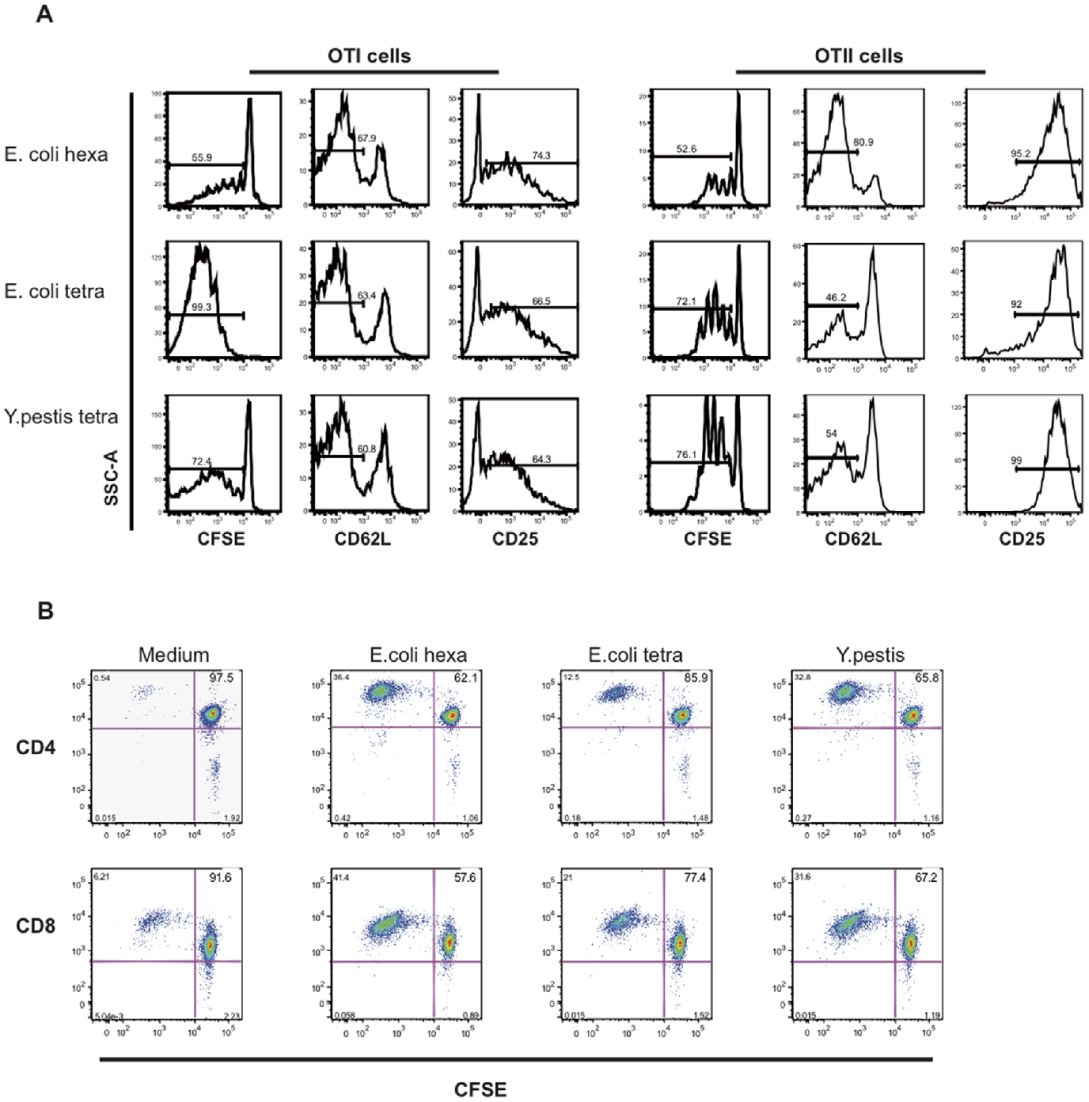
LPS with acylation defects induce functional mouse and human dendritic cells. BMDC were incubated overnight with OVA and activated for 8 h with different LPS. Stimulated DC were co-cultured with T cells from OT-I and OT-II *Rag-2^−/−^* mice (A). The proliferation of OT-I and OT-II T cells was assessed after 3 days of co-culture by CFSE decrease. For T cell activation assays, the expression of surface markers such as CD25 and CD62L was analyzed by flow cytometry. At least 3 independent experiments were performed and one representative is shown. (B) CFSE-labeled allogeneic naïve CD4^+^ T and CD8^+^ T cells were co-cultured with activated mDC for 7 days. Cell division was tested by measuring CFSE-dilution Experiments were performed on 4 different donors. Data for one representative experiment are shown.

We next studied the antigen presentation capacity of tetra-acyl LPS-treated DC and their ability to promote T cell responses (Figure 6). We used transgenic mice that express either a TCR specific for the MHC class-I restricted OVA (OT-I *Rag-2* ^−/−^) or a TCR specific for the MHC class-II restricted OVA (OT-II *Rag-2* ^−/−^). BMDC incubated in either medium alone or medium containing ovalbumin (OVA) were activated by different LPS and co-cultured with OTI (CD8^+^) and OTII (CD4^+^) T cells for 3 days (Figure 6A). Basal level of T cell responses was determined. BMDC incubated with LPS alone or OVA alone could not induce any T cell response (data not shown). However, BMDC incubated with OVA and activated by different LPS efficiently induced antigen-specific CD8^+^ and CD4^+^ T cell responses (Figure 6A). DC activated by tetra-acyl LPS induced a higher OTI and OTII T cell proliferation than cells treated by hexa-acyl LPS (Figure 6A). DC stimulated by tetra-acyl and hexa-acyl LPS were able to trigger T cell activation characterized by a CD25 up-regulation and a CD62L down-regulation. However hexa-acyl LPS-treated BMDC led to a higher down-regulation of CD62L by OT II T cells than those treated with tetra-acyl LPS (Figure 6A). Altogether, these data show that BMDC induced by LPS with acylation defects are able to efficiently promote antigen presentation and induce CD8^+^ and CD4^+^ T cell responses.

We then investigated the functional properties of human DC stimulated with LPS variants (Figure 6B). Human blood myeloid DC (mDC) activated by the different LPS were able to induce the proliferation of allogeneic naïve CD4^+^ and CD8^+^ T cells, although to a lower level for *E. coli* tetra-acyl LPS compared to other LPS (Figure 6B). Tetra-acyl LPS from *Y. pestis,* which contains small amounts of hexa-acyl LPS had a stronger capacity to trigger T cell responses than LPS purified from *E. coli* (*msbB*-, *htrB*-) double mutant (devoid of hexa-acyl LPS) (Figure 6B, Table 1). These results show that tetra-acyl LPS-treated DC are able to promote CD4^+^ and CD8^+^ T cell responses both in mouse and human models.

**Figure 7:**
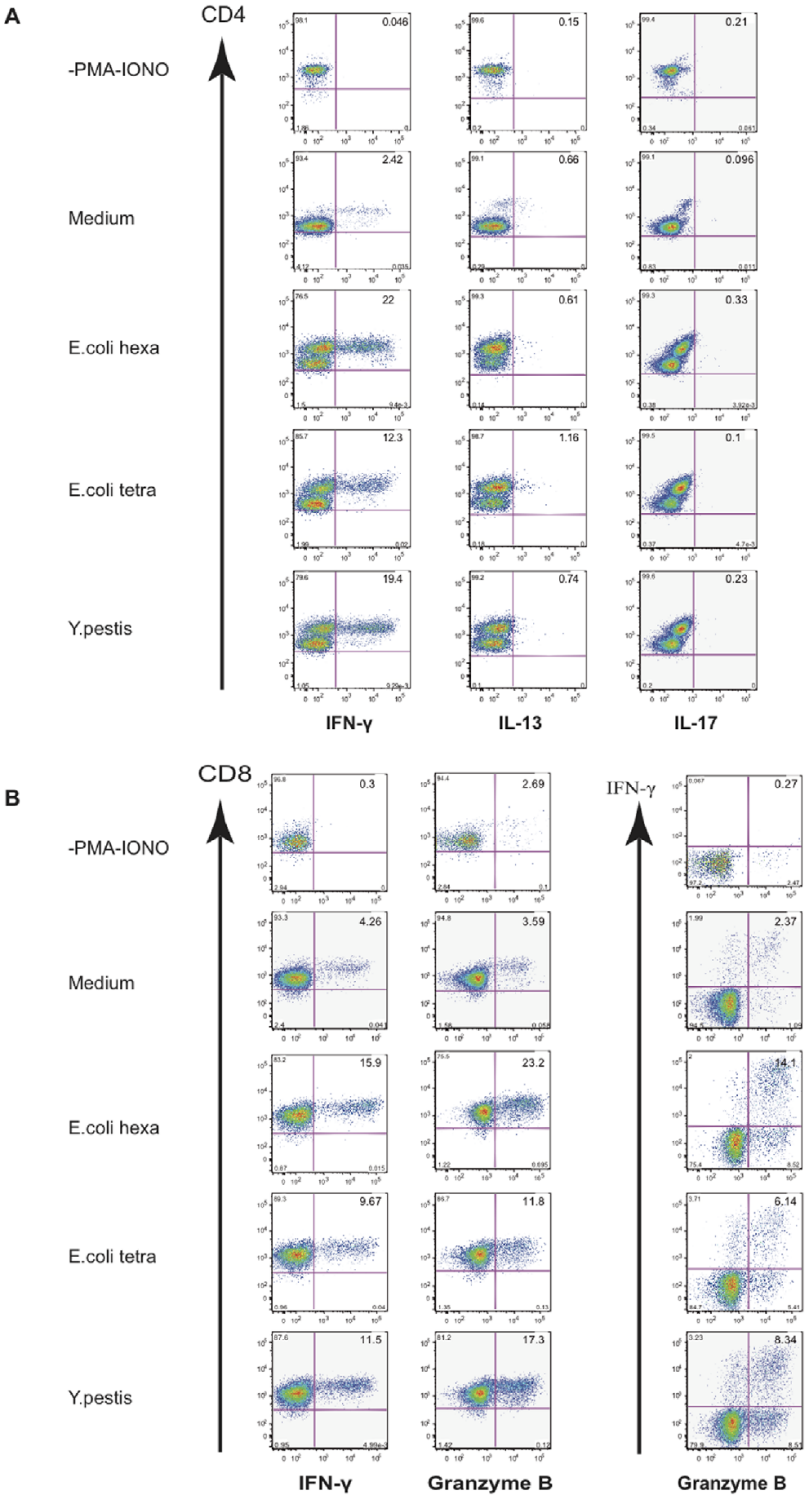
Tetra-acyl LPS induce effector molecules synthesis by human T cells. Human blood mDC were activated overnight either by medium or LPS variants and co-cultured with allogeneic naïve CD4^+^ T and CD8^+^ T cells. After 7 days, cells were incubated 6 h with PMA/Ionomycine in the presence of Brefeldin A. The intracellular levels of IFN-γ, IL-13 and IL-17 in CD4^+^ T (A) and IFN-γ and Granzyme B in CD8^+^ T cells (B) were analysed by flow cytometry. Experiments were performed on 4 different donors. Data for one representative experiment are shown.

We then characterized the effector T cells induced by LPS-treated mDC (Figure 7). Cells were stimulated with PMA/Ionomycin and stained for intracellular IFN-γ (T_H1_ response), IL-13 (T_H2_ response) and IL-17 (T_H17_ response). mDC stimulated either by hexa- or tetra-acyl LPS polarized allogeneic naïve CD4^+^ T cells into IFN-γ-expressing T_H1_ cells (Figure 7A). CD4^+^ T cells co-cultured with either hexa-acyl LPS-activated mDC or tetra-acyl-activated mDC did not express IL-13 or IL-17 (Figure 7A). mDC stimulated by tetra-acyl LPS were also able to induce IFN-γ and Granzyme B synthesis in CD8^+^ T cells (Figure 7B). However, we observed lower levels of IFN-γ and Granzyme B production with LPS purified from *E. coli* MLK (*msbB*-, *htrB*-) double mutant compared to other LPS (Figure 7).

These data indicate that DC activated by either hexa-acyl or tetra-acyl LPS induce T_H1_ responses and activate CD8^+^ T cells.

### In Contrast to Murine BMDC, Tetra-acyl LPS Activate Human DC to Induce T_reg_ cells

DC with MHC II^high^, co-stimulation^high^, pro-inflammatory cytokines^ low^ phenotype are referred in the literature as semi-mature. It has been shown that these cells are able to trigger the differentiation of regulatory T cells (T_reg_) [17]. We thus evaluated whether mouse BMDC activated by tetra-acyl LPS displaying a semi-mature phenotype were capable of generating T_reg_ cells characterized by the expression of the transcriptional factor Foxp3 and a high CD25 expression at their cell surface. When maintained on a *Rag-2* ^−/−^ background, transgenic mice that express a TCR specific for I-A^b^-OVA complexes (OT-II *Rag-2* ^−/−^ mice) contain only conventional (Foxp3^−^) CD4^+^ T cells in their periphery, a situation that facilitates the measurement of their conversion into T_reg_ cells [18]. Such conversion requires I-A^b+^ DC and the presence of the OVA-derived peptide specifically recognized by OT-II CD4^+^ T cells (Figure S4). It also depends on the secretion by the antigen-presenting DC of TGF-β [18]. Accordingly, BMDC stimulated with different LPS variants were incubated with OT-II *Rag-2* ^−/−^ T cells in the presence of the OVA or OVA_257–264_ peptide (0.06 µg/mL), with or without TGF-β (Figure S4). We could observe that OVA and peptide-pulsed BMDC were both capable of inducing the activation of OT-II *Rag-2* ^−/−^ CD4^+^ T cells as measured by CD25 expression (Figure S4). However, DC stimulation either by tetra-acyl or hexa-acyl LPS did not trigger T_reg_ responses in mouse BMDC (Figure S4A). The addition of exogenous TGF-β to the culture did not confer to LPS-activated DC the ability to generate T_reg_ cells (Figure S4B).

**Figure 8:**
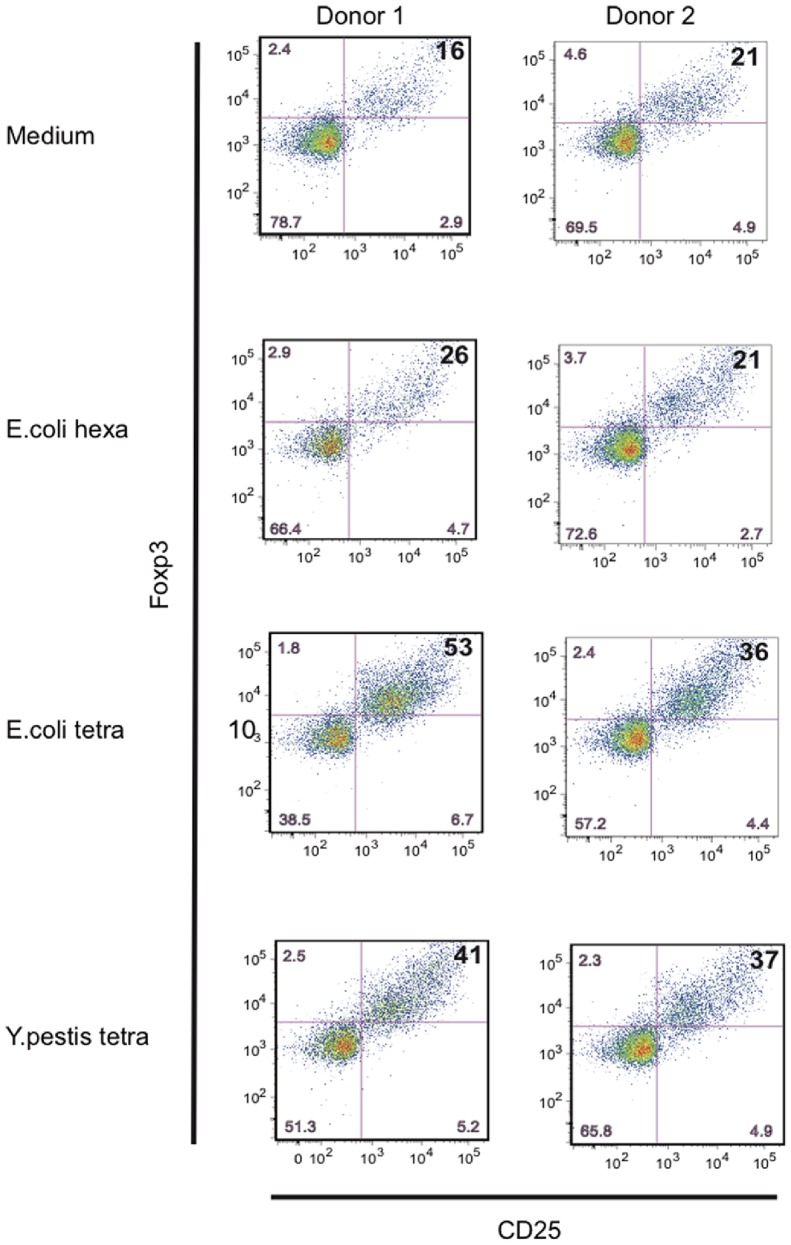
LPS with acylation defects activate human mDC to induce regulatory T cells. Human blood mDC were activated overnight either by medium or different LPS and co-cultured with allogeneic naïve CD4^+^ T cells. After 7 days, cells were incubated 6 h with PMA/Ionomycine in the presence of Brefeldin A. Foxp3 and CD25 expression was analysed in CD4^+^ T cell population. Experiments were performed on 4 different donors. Data for 2 representatives are shown.

We then studied the capacity of human mDC activated by tetra-acyl LPS to induce T_reg_ cells. Human DC activated by LPS variants were co-cultured with allogeneic naïve CD4^+^ T cells and T_reg_ population was analysed by flow cytometry (Figure 8). We could observe that mDC activated by tetra-acyl LPS induced a higher T_reg_ population characterized by the expression of Foxp3 and a high CD25 expression at the cell surface (Figure 8). This activation profile could be due to the fact that human DC activated by different forms of tetraacyl LPS, including the synthetic Lipid IVa display an intermediate profile of DC maturation (as shown here for IL-4 DC in Figure S5) then leading to T_reg_ proliferation.

## Discussion

The innate immune system possesses various mechanisms to detect and facilitate host responses to microbial components such as LPS [19]. It has been described that each change in chemical composition of LPS causes a dramatic decrease of its activity down to a complete loss of endotoxicity [6]. Different cell types, mainly human and mouse monocytes/macrophages have been used to study LPS structural requirements for its immunostimulatory properties. However, to determine the endotoxic activity of enterobacterial LPS, previous studies have mainly concentrated on cytokine production. Consequently, a decrease in IL-8, IL-6 and TNF-α secretion by cells stimulated with LPS harboring acylation defects has been considered as a lack of immunogenicity or a defect of pro-inflammatory signaling [9], [10], [20]. In contrast, we show here that LPS with acylation defects efficiently induce a potent activation of TLR4-dependent signaling in mouse and human DC that leads to a strong cytokine synthesis, which in turn triggers the activation of the proteasome machinery. The consequence is the degradation of intracellular pro-inflammatory cytokines and consequently the decrease of their secretion. This hypothesis corroborates previous results, which showed a decrease of cytokine secretion in tetra-acyl LPS-treated macrophages [8], [9], [10], [20].

The difference in the activation potential of LPS variants in terms of cytokine secretion could affect the output of the DC immune response. DC activated by tetra-acyl LPS triggered CD4^+^ T and CD8^+^ T cell responses both in mouse and human DC. However, human DC activated by LPS with acylation defects displayed a semi-mature phenotype and induced T_reg_ responses. There could be several mechanisms by which tetra-acyl LPS interact with human DC to elicit distinct types of T_H_ responses. Functional differences between the different subsets of human myeloid DC could be one possible explanation. Two main populations of circulating DC termed myeloid (mDC) and plasmacytoid (pDC) were identified in the blood of healthy donors. Additional distinctions can be made within the mDC subset with CD1c^+^CD141^−^ mDC1, CD1c^−^CD141^+^ mDC2 and CD16^+^ mDC [21]. It has been shown that mDC1 and mDC2 differ for the expression of surface markers, cytokine production profile and the differentiation of T_H_ responses. When co-cultured with purified human peripheral blood cells, mDC1 produce IL-12 and favor T_H1_ differentiation, while mDC2 produce high levels of IL-10 and direct the differentiation of T_H2_. Moreover, the identification of numerous phenotypic and functional differences among pulmonary mDC1 and mDC2 suggests a possible preferential role for mDC2 in regulating immunity and disease pathogenesis in the respiratory tract distinct from that of mDC1. Distinct roles in host immunity for each human DC were previously shown [21], [22], [23], [24]. For instance, the human CD1c^−^CD141^+^ mDC2 subset is the functional equivalent of mouse CD8α^+^ DC, capable of cross presentation of exogenous antigens. Regarding their capacity to secrete IL-10, mDC2 might also induce T_reg_ populations.

T_reg_ are key players in the immune regulation, particularly in tolerance. This cell population plays a crucial role in suppressing immune responses to self-antigens and in preventing autoimmune diseases [25], [26]. Evidence is emerging that T_reg_ can control immune responses to pathogens. They are beneficial to the host through limiting the immunopathology associated with anti-pathogen immune responses and enabling the development of immune memory. However, pathogens can exploit T_reg_ to subvert the protective immune responses of the host in order to survive and establish a chronic infection [27], [28]. Microbes have evolved strategies for programming DC to induce T_reg_ in order to maintain immune homeostasis that controls unbridled host immunity [4], [27]. For example, filamentous hemagglutinin (FHA) from the bacteria *Bordetella pertusis* induces DC to provide IL-10 and prime T_reg_. Moreover, *Yersinia pestis* is known to activate DC by means of the dimer of TLR2 and TLR6 to induce T_reg_ [29].

There is growing evidence that the induction of tolerance is not restricted to immature DC. Within the tolerogenic pool of DC, a third population is proposed, called semi-mature [17]. This new subset or developmental stage of DC is distinguished as mature by their surface marker analysis (MHC II^high^ and co-stimulation^ high^). However, semi-mature DC do not release high level of pro-inflammatory cytokines, such as IL-1β, IL-6, TNF-α or IL-12p40 or IL-12p70. IL-10 production by semi-mature DC has been described, but it is not an absolute requirement for T_reg_ differentiation [17]. Inducers of DC semi-maturation can be lactobacilli from the gut flora [30], intranasally applied OVA [31], apoptotic cells [32], *Bordetella pertussis* FHA [33] or TNF-α [34]. Here we show that, structural modifications of LPS are able to induce semi-mature human and mouse DC characterized by MHC-II^high^, co-stimulation^high^, pro-inflammatory cytokines^ low^ phenotype. In the human model, these semi-mature DC induce high levels of T_reg_ cells.

In conclusion, we describe a new mechanism, which regulates the pro-inflammatory cytokine decrease in cells activated by LPS with acylation defects. We propose that cell stimulation by tetra-acyl LPS trigger the activation of the proteasome machinery. This leads to the degradation of intracellular pro-inflammatory cytokine levels and consequently to a decrease of their secretion. Our results provide new insights into the understanding of early steps of endotoxin action and suggest that structural modifications of LPS could represent an important strategy for pathogens to subvert adaptive immunity by T_reg_ cell induction in order to survive.

## Supporting Information

Figure S1 **LPS structure effect on mouse BMDM activation.** Mouse BMDM were incubated with medium, *E. coli* hexa-acyl LPS (dark blue), *E. coli* tetra-acyl LPS (purple) or *Y. pestis* tetra-acyl LPS (light blue). Secretion levels of TNF-α were determined by ELISA after 8 h and 24 h of cell activation. Data represent means ± standard errors of at least 4 independent experiments. **p<0.01.
(EPS)

Figure S2 **Tetra-acyl LPS induce a TLR4-dependent DC activation.** BMDC from wild type and *Tlr4* ^−/−^ mice (A) or *Tlr2* ^−/−^ mice (B) were stimulated for 8 h and 24 h with medium (grey) or *E. coli* hexa-acyl LPS (dark blue), *E. coli* tetra-acyl LPS (purple) or *Y. pestis* tetra-acyl LPS (light blue) or Pam2CSK4 (brown). TNF-α secretion was measured by ELISA. Data represent means ± standard errors of at least 3 independent experiments, ***p<0.001, **p<0.01.
(EPS)

Figure S3 **LPS effect on mouse NF-κB translocation in mouse BMDM.** Mouse BMDM were incubated with medium (grey), *E. coli* hexa-acyl LPS (dark blue), *E. coli* tetra-acyl LPS (purple) or *Y. pestis* tetra-acyl LPS (light blue). (A) NF-κB translocation was analyzed by confocal microscopy in cells activated with different LPS for 15 min, 30 min, 1 h and 2 h. Cells were fixed and stained for NF-κB subunit p65/RelA (in red). The percentage of BMDM with translocated NF-κB into the nucleus was quantified (B). Data represent means ± standard errors of at least 4 independent experiments, **p<0.01.
(EPS)

Figure S4 **BMDC capacity to trigger T_reg_ cell differentiation.** BMDC stimulated with different LPS variants were incubated with OT-II *Rag-2* ^−/−^ T cells in the presence of the OVA, OVA_257–264_ peptide (0.06 µg/mL) with or without TGF-β. After 5 days of culture, T cells were analyzed for the expression of Foxp3 and of CD25. Numbers in outlined areas indicate percentage of cells. Results for hexa-acyl and tetra-acyl *E. coli* LPS are shown. Data similar to tetra-acyl *E. coli* LPS are observed while BMDC are stimulated with tetra-acyl *Y. pestis* LPS. Data are representative of 3 independent experiments.
(EPS)

Figure S5 **Human IL-4 DC stimulation properties in the presence of** ***E. coli*** **LPS analogs and** ***Y. pestis*** **LPS.** IL-4 DC were stimulated for 72 h with medium, hexa-acyl *E. coli* LPS, tetra-acyl *E. coli* LPS, synthetic Lipid IVa and *Y. pestis* at 20 ng/ml. Cell culture supernatants were kept for cytokine measurement (IL-6, IL-10 and TNFα) by Luminex (A). Surface expression of HLA-DR, CD80 and CD86 was analyzed by flow cytometry (B) Experiments were performed on 4 different donors. Data for one representative donor are shown.
(TIF)
