# Supplementary figures and images for: Lipopolysaccharides with Acylation Defects Potentiate TLR4 Signaling and Shape T Cell Responses
Source: PLoS One. 2013 Feb 4;8(2):e55117. doi: 10.1371/journal.pone.0055117 (PMC3563657; doi:10.1371/journal.pone.0055117)

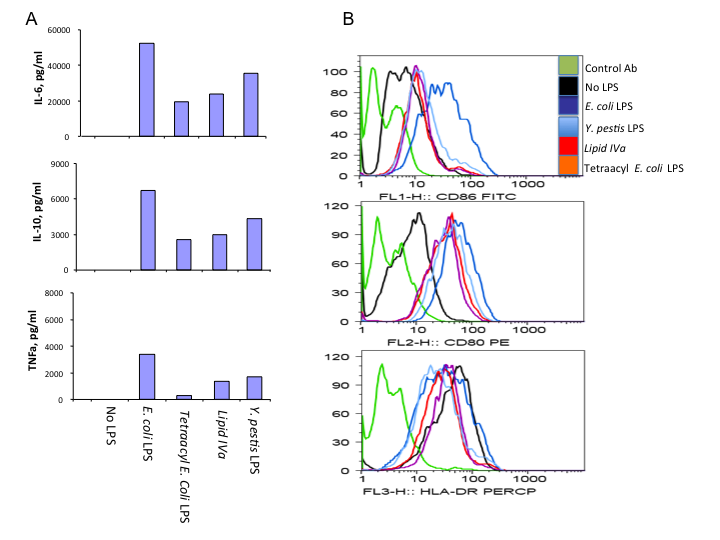

Supplement: Figure S5 — Human IL-4 DC stimulation properties in the presence of E. coli LPS analogs and Y. pestis LPS. IL-4 DC were stimulated for 72 h with medium, hexa-acyl E. coli LPS, tetra-acyl E. coli LPS, synthetic Lipid IVa and Y. pestis at 20 ng/ml. Cell culture supernatants were kept for cytokine measurement (IL-6, IL-10 and TNFα) by Luminex (A). Surface expression of HLA-DR, CD80 and CD86 was analyzed by flow cytometry (B) Experiments were performed on 4 different donors. Data for one representative donor are shown. (TIF) [file pone.0055117.s005.tif]
